# Characterization of Antibacterial and Hemolytic Activity of Synthetic Pandinin 2 Variants and Their Inhibition against *Mycobacterium tuberculosis*

**DOI:** 10.1371/journal.pone.0101742

**Published:** 2014-07-14

**Authors:** Alexis Rodríguez, Elba Villegas, Alejandra Montoya-Rosales, Bruno Rivas-Santiago, Gerardo Corzo

**Affiliations:** 1 Departamento de Medicina Molecular y Bioprocesos, Instituto de Biotecnología, Universidad Nacional Autónoma de México, Cuernavaca Morelos, México; 2 Centro de Investigación en Biotecnología, Universidad Autónoma del Estado de Morelos, Cuernavaca, Morelos, México; 3 Medical Research Unit-Zacatecas, Mexican Institute of Social Security, UIMZ-IMSS, Zacatecas, Mexico; University Hospital Schleswig-Holstein, Campus Kiel, Germany

## Abstract

The contention and treatment of *Mycobacterium tuberculosis* and other bacteria that cause infectious diseases require the use of new type of antibiotics. Pandinin 2 (Pin2) is a scorpion venom antimicrobial peptide highly hemolytic that has a central proline residue. This residue forms a structural “*kink*” linked to its pore-forming activity towards human erythrocytes. In this work, the residue Pro14 of Pin2 was both substituted and flanked using glycine residues (P14G and P14GPG) based on the low hemolytic activities of antimicrobial peptides with structural motifs Gly and GlyProGly such as magainin 2 and ponericin G1, respectively. The two Pin2 variants showed antimicrobial activity against *E. coli*, *S. aureus*, and *M. tuberculosis*. However, Pin2 [GPG] was less hemolytic (30%) than that of Pin2 [G] variant. In addition, based on the primary structure of Pin2 [G] and Pin2 [GPG], two short peptide variants were designed and chemically synthesized keeping attention to their physicochemical properties such as hydrophobicity and propensity to adopt alpha-helical conformations. The aim to design these two short antimicrobial peptides was to avoid the drawback cost associated to the synthesis of peptides with large sequences. The short Pin2 variants named Pin2 [14] and Pin2 [17] showed antibiotic activity against *E. coli* and *M. tuberculosis*. Besides, Pin2 [14] presented only 25% of hemolysis toward human erythrocytes at concentrations as high as 100 µM, while the peptide Pin2 [17] did not show any hemolytic effect at the same concentration. Furthermore, these short antimicrobial peptides had better activity at molar concentrations against multidrug resistance *M. tuberculosis* than that of the conventional antibiotics ethambutol, isoniazid and rifampicin. Therefore, Pin2 [14] and Pin2 [17] have the potential to be used as an alternative antibiotics and anti-tuberculosis agents with reduced hemolytic effects.

## Introduction

Cationic Antimicrobial Peptides (CAMPs) are components of the biological defense system of microorganisms, plants, animals and humans [1]–[3]. A large number of CAMPs have been characterized in invertebrates, especially from the phylum Arthropoda [4]–[6], and in vertebrates from the class Amphibia [7], [8]. CAMPs with alpha-helical conformation share some common characteristics such as antimicrobial activities at low micromolar concentrations and alpha-helix conformation in hydrophobic environments [9]–[11]. They have potent antibacterial activities that made them promissory candidates to develop novel antibiotics because of their broad-spectrum activity towards multi-resistant pathogenic Gram-positive and Gram-negative bacteria, as well as towards clinically important yeasts such as *Candida albicans* [12]–[14].

Pin2 (FWGALAKGALKLIPSLFSSFSKKD, see table 1) is a 24-residue alpha-helical antimicrobial peptide characterized from the venom of the African scorpion *Pandinus imperator*, this peptide has antimicrobial activities towards Gram-positive and Gram-negative bacteria in the micromolar range; however, it shows hemolytic activity at similar concentrations [15]. High-resolution structure of Pin2, determined by NMR, showed that the peptide is essentially alpha-helical with a proline, which induces a structural “*kink*” in the central part of its structure [15]. The proline “*kink*” is a structural characteristic of some CAMPs that confer them high pore-forming abilities. Clear examples of such peptides are melittin [16], alamethicin [17] and pardaxin [18]. In those peptides the presence of a proline “*kink*” was correlated to a high antimicrobial activity but unfortunately also showed high hemolytic activities [17], [19]–[21]. The elimination of the proline or its substitution in their primary structure of these peptides had different effects on their secondary structures and biological activities. For example, the substitution of the P14 for alanine in the antimicrobial peptides melittin and pardaxin increase their hemolytic and antimicrobial activities, respectively, as result of an increment in the helicity grade of such antimicrobial analogs [19]. On the other hand, CAMPs such as Magainin 2 from the African frog *Xenopus laevis*, Oxypinins from the wolf spider *Oxyopes takiobus* and Ponericins G1 from the ponerin ant, *Pachycondyla goeldii*, showed antimicrobial activities with low cytotoxic effects towards erythrocytes [22]–[24]. These peptides have different amino acid motifs in the central region of their primary structures. For example, magainin 2 has a single glycine in the middle of its structure, Oxypinin 2b (Oxki2b) has a GlyValGly motif, and Ponericin G has glycine residues flanking the central proline, a GlyProGly motif. Furthermore, cecropin and melittin synthetic hybrids in which the proline of melittin was changed by Cecropin residues showed no hemolytic activities in contrast to the parental peptide [25]. Likewise, the substitution of the proline residue (P14) in Pin2 for the residues Val, GlyVal, ValGly or GlyValGly reduced the hemolytic activity of Pin2 without any significant changes in its antimicrobial activity [26].

**Table 1:** Physicochemical properties of Pin2 and Pin2 variants.

							Molecular Weight (Da)	
Peptide	Sequence	GRAVY	AMF	μHrel	Q	RT (min)	Theoretical	Experimental^§^
**Pin2**	FWGALAKGALKLIPSLFSSFSKKD	0.329	0.4283	0.48	+3	30.6	2612.1	2612.0
**Pin2 [G]**	FWGALAKGALKLIGSLFSSFSKKD	0.379	0.4296	0.49	+3	40.1	2572.0	2572.0
**Pin2 [GPG]**	FWGALAKGALKLIGPGSLFSSFSKKD	0.273	0.4369	0.28	+3	27.6	2726.2	2727.0
**Pin2 ** [14]	FWGLKGLKKFSKKL	−0.357	0.4271	0.51	+5	18.5	1680.1	1680.0
**Pin2 ** [17]	FWGLKGLKGPGKFSKKL	−0.435	0.4453	0.35	+5	17.2	1891.3	1891.3

**GRAVY, Sequence Grand average of hydropathicity, calculated using the Expasy ProtParam tool (** http://web.expasy.org/protparam/ **), according to Kyte and Doolittle, 1982** **[44]** **.**

**AMF, Average Molecular Flexibility values were calculated according to Liu** ***et al*** **., 2008** **[45]** **.**

**μHrel, Relative Hydrophobic Moment, a value of 0.5 thus indicates that the peptide has about 50% of the maximum possible amphipathicity. Calculated using HydroCalc (** http://www.bbcm.univ.trieste.it/~tossi/HydroCalc/HydroMCalc.html **).**

**Q, Net charge.**

**RT, Retention Time in minutes.**

**(§)Mass Spectrometry, ESI-MS (Finnigan LCQ^DUO^ ion trap mass spectrometer, San José, CA, USA).**

In this work, based on the low hemolytic activities shown by the antimicrobial peptides magainin 2 from *X. laevis* and Ponericin G1 from *P. goeldii*, two synthetic variants of Pin2, Pin2 [G] and Pin2 [GPG] were chemically synthesized with the aim to reduce the hemolytic activity and preserve the antibiotic activities of Pin2. In addition, two short variants of Pin2, with 14 and 17 residues, respectively, were designed and chemically synthesized with the aim to continue reducing their hemolytic but keeping their antimicrobial activities as well as to reduce the number of residues to have a low cost CAMP. Here these antimicrobial peptide variants are proposed as potential antibiotics for the clinical treatment of pathogenic bacteria including *Mycobacterium tuberculosis*.

## Materials and Methods

### Ethics statement

Approvals to conduct experimental protocols to study hemolysis on human red cells were approved by the Bioethics Committee of the Biotechnology Institute, where this work was done. Human red cells were from volunteer Gerardo Corzo, who signed the informed consent for this study and is also author of this report.

### Microorganisms

The bacterial strains used were *Escherichia coli* (ATCC 25922) and *Staphylococcus aureus* (ATCC 25923). They were purchased directly from the American Type Culture Collection (ATCC) through The Global Bioresource Center. *Mycobacterium tuberculosis* H37Rv (ATCC 27294) [27] and *M. tuberculosis* muti-drug resistant strain (MDR) [28] were from the Medical Research Unit-Zacatecas belonging to the Mexican Institute of Social Security.

### Solid phase peptide synthesis

Pin2 and Pin2 variants were chemically synthesized by a solid-phase method using the Fmoc methodology on an Applied Biosystems 433A peptide synthesizer. Fmoc-Asp(otBu) or Fmoc-Leu(otBu)-Wang resins were used to provide a free carboxyl at the C-terminus of the Pin2 and its variants. Cleavage and deprotection of peptides, from resins and from protecting side chain groups, were performed using a chemical mixture composed of 1 g crystalline phenol, 0.2 g imidazole, 1 mL thioanisol, 0.5 mL 1,2-ethanedithiol in 20 mL trifluoroacetic acid (TFA). The resin was removed by filtration, and the deprotected peptides in solution were precipitated using cold ethyl ether. The precipitated peptides were washed twice with cold diethyl ether to remove remaining scavengers and protecting groups. Each crude synthetic peptide was then dissolved in a 10% aqueous acetonitrile solution and separated by reverse phase HPLC (RP-HPLC) on a semipreparative C_18_ column (10×250 mm, Vydac, USA). Cationic exchange chromatography and C_18_ analytical RP-HPLC were further used to purify the synthetic peptides, after all purification steps, the final purity of the peptides was higher than 95%. The molecular masses of the synthetic peptides were obtained by mass spectrometry using a LCQ DUO ion trap mass spectrometer (Finnigan, San Jose, USA) with and ESI source from 2.1 to 3.1 kV.

### Antimicrobial assays

Minimal inhibitory concentrations (MIC) and growth inhibition curves were obtained using pure peptides in the presence of bacteria using two different methods, agar diffusion susceptibility assays and broth microdilution assays in accordance to the procedures from the Clinical and Laboratory Standards Institute (CLSI, http://www.clsi.org).

The agar diffusion susceptibility assay was performed using 10 mL of Mueller-Hinton agar (MHA) underlay on a Petri dish plate, while at the same time, 0.1 mL aliquot of a mid-logarithmic-phase (1×10^8^ CFU/mL in MHB with A_625nm_ = 0.5) culture, was inoculated in a sterile tube containing 9.9 mL of non solidified MHA and mixed. The content of the tube was overlaid in the previously poured MHA Petri dish. Then 5 µL aliquots of a diluted antimicrobial peptide at 300, 100, 80, 50, 37.5, 25, 18.8, 12.5, 6.25, 3.1 and 1.6 µM were subsequently loaded into the overlay gel. Samples were incubated overnight at 37°C. The antimicrobial activity was determined by measuring clear zone diameters or halos observed around each peptide concentration in cultured MH Petri dishes. Peptide MICs were defined as the lowest peptide concentration with a clear zone halo.

Broth microdilution assays were performed using stock solutions of Pin2 [G], Pin2 [GPG], Pin2 [14] or Pin2 [17] antimicrobial peptide diluted serially from 25 to 0.4 µM to a final volume of 200 µL, placed in polypropylene microtubes and vacuum dried. Next a volume of 200 µL aliquots of the bacterial suspension (1×10^5^ CFU/mL) in MHB was dispensed into each of the polypropylene microtubes and mixed with the diluted antimicrobial peptide. Then each was transferred into a well of a 96-well microtiter plate and bacterial growth was evaluated by measuring absorbance every 2 h until 10 h of incubation time at 37°C. The optical density (OD) of each well was measured at 625 nm in an ELISA reader (BioRad, model 450, Hercules, CA, USA). The positive control contained only the bacterial suspension, and the negative control contained only sterile culture medium. The resulting MICs were defined as the lowest peptide concentration that showed zero visible growth or absence of growth, that is growth inhibition (100%). The minimal inhibitory concentrations (MIC) values were the mean result of three independent experiments.

### Mycobacterium tuberculosis assays

The Resazurin microtitre assay plate (REMA) method was conducted to determine the *M. tuberculosis* susceptibility to the action of Pin2 and the different Pin2 variants studied in this work. Resazurin is an oxidation–reduction indicator and has been used to assess viability, bacterial contamination and to test for antimicrobial activity [29]. The *M. tuberculosis* H37Rv (ATCC 27294) and a clinically isolated multidrug resistant (MDR) strain were used in these experiments. The REMA plate method was performed in 7H9 medium containing 10% of OADC (oleic acid, albumin, dextrose and catalase) (Becton–Dickinson, Sparks, MD, USA). Resazurin sodium salt powder (Sigma) was prepared at 10% (wt/vol) in distilled water and filter-sterilized (0.22 µm). Two-fold serial dilutions of each synthetic peptide dissolved in 100 µL of 7H9-OADC culture medium were added directly in 96-well plate at concentrations ranging from 96.2 to 0.3 µg/mL. Ethambutol and rifampicin were used as positive controls. The *M. tuberculosis* inoculum was prepared from a 14-day logarithmic phase culture and adjusted to 1 of the Mcfarland scale (0.76 OD, 600 nm) with 7H9-OADC culture medium, and then diluted to 1∶20. Then, 100 µL of these inoculums were added to each well on the plate (final volume 200 µL). The plates were covered with proper lids and incubated at 37°C in a normal atmosphere. After 5 days of incubation, 20 µL of the Resazurin solution were added to each control well only, and incubated for 24 h at 37°C for color development. If color was developed as expected (pink for microorganism's control growth and blue for sterility control), 20 µL of the Resazurin solution were added into each well containing the peptides at different concentrations. After 24 h, a change from blue color to pink color indicated the reduction of Resazurin and therefore bacterial growth. The minimal inhibitory concentration (MIC) was defined as the lowest peptide concentration that prevented the reduction of Resazurin and therefore a color change from blue to pink. Previous studies suggest that some host defense peptides may induce dormancy or a bacteriostatic state in *M. tuberculosis* [30]. To examine this, 10 µL from the lowest concentration that did not reduce Resazurin were serially diluted and seeded onto 7H10 agar plates supplemented with Middlebrook OADC enrichment media and incubated for at least 21 days at 37°C, to observe if *M. tuberculosis* growth reestablishment occurred. All antimicrobial tests were conducted in triplicate.

### Hemolytic assays

Hemolytic activity was determined by incubating suspensions of human red blood cells with serial dilutions of each selected peptides. Red blood cells were rinsed several times in PBS by centrifugation for 3 min at 3,000 *g* until the OD of the supernatant reached the OD of the control (PBS only). Red blood cells were counted by a hemocytometer and adjusted to 7.7×10^6^±0.3×10^6^ cells/mL. Red blood cells were then incubated at room temperature for 1 h in 10% Triton X-100 (positive control), in PBS (blank), or with amphipathic peptides at concentrations of 0.4, 0.8, 1.6, 3.1, 6.2, 12.5 and 25 µM, only for Pin2 [14] and Pin2 [17] the 50 and 100 µM concentrations were evaluated. The samples were then centrifuged at 10,000 *g* for 5 min, the supernatant was separated from the pellet, and its absorbance measured at 570 nm. The relative optical density compared to that of the suspension treated with 10% Triton X-100 was defined as the percentage of hemolysis.

### Circular Dichroism (CD) measurements

The CD experiments were recorded on a Jasco model J-720 spectropolarimeter (Tokyo, Japan). The different spectra were measured from 260 to 190 nm on samples in water, 20, 40 and 60% trifluoroethanol (TFE), at room temperature, with a 1-mm-pathlength cell. Data were collected at 1 nm with a scan rate of 100 nm/min and a time constant of 0.5 s. The concentration of each peptide was 150 µg/mL Data were the average of five separate recordings and were analyzed on line by the software K2d (http://www.embl.de/~andrade/k2d.html) [31], [32].

### Statistical analysis

The experimental values represent means ± standard deviations. The hemolytic constants (IC_50_) were obtained using a non-linear regression where the data was fit to a Boltzmann sigmoid equation. To determine statistically significant differences, an analysis of variance (ANOVA) followed by post hoc testing using the Tukey's method was performed using the software package Prism 4 (GraphPad, Inc., USA). Statistical significances were accepted at p<0.05.

## Results

### Design of Pin2 [G], Pin2 [GPG] and their short variants

**Figure 1:**
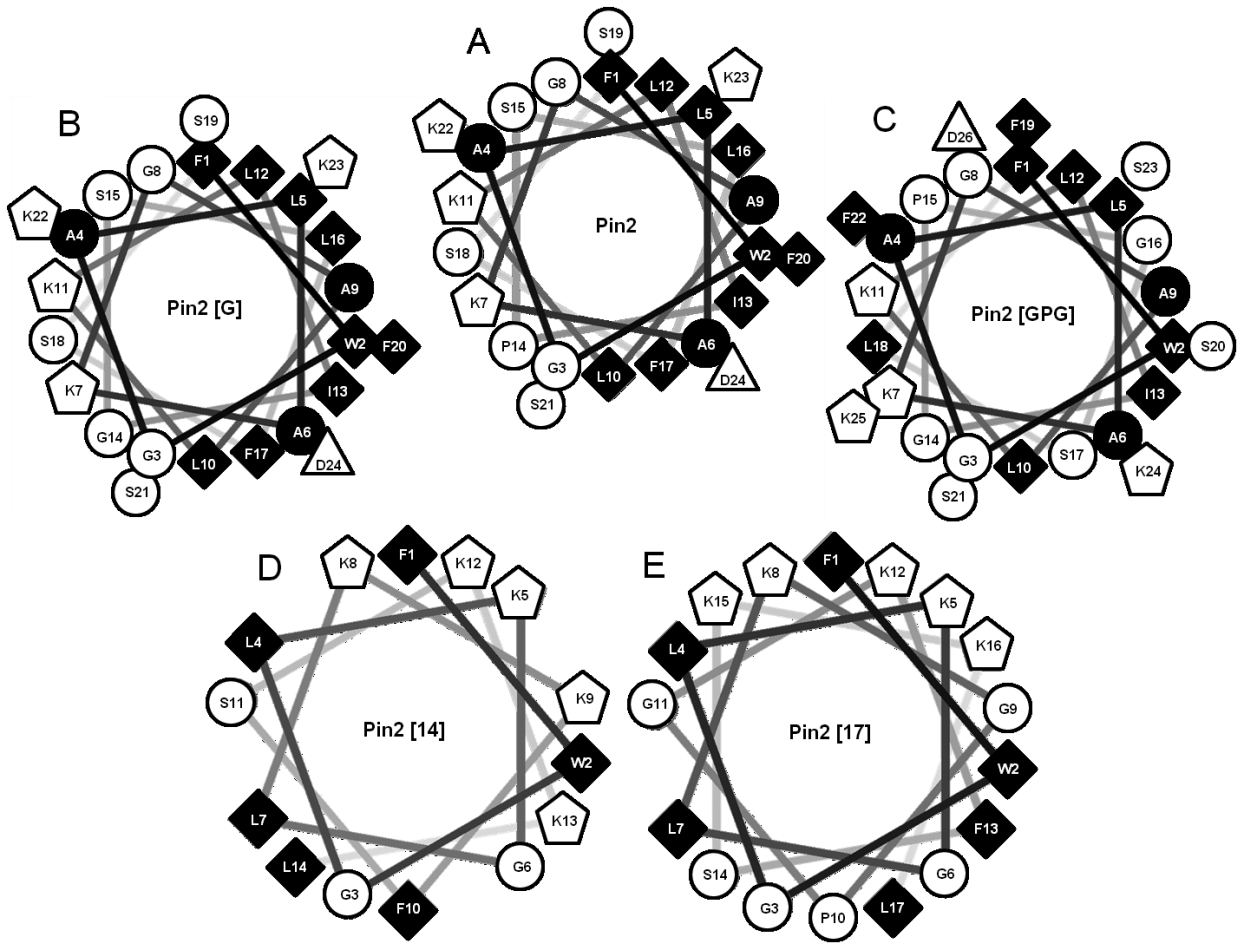
Helical wheel diagrams of Pin2 and Pin2 variants. Helical wheels were prepared by the software Helical Wheel Projections [56]. The hydrophobic residues are colored in black, hydrophilic and neutral residues are colored in white. A. Pin2, B. Pin2 [G], C. Pin2 [GPG], D. Pin2 [14] and E. Pin2 [17].

The physicochemical parameters of CAMPs such as the net charge, the amphipathic character and the hydrophobicity could be modified to improve their antimicrobial properties, inclusive cell membrane selectivity [33]–[35]. However, still there are several drawbacks for the development of CAMPs as therapeutic agents, among them the synthesis cost for their production is one of the most important [36]–[38]. Therefore in this work, we attempt to design first low hemolytic CAMPs and subsequently reduce the number or residues. Hence, the variants of Pin2 [G] and Pin2 [GPG] were designed to reduce their hemolytic activities but to preserve their antibiotic activities. Here, the proline residue (P14) was the target as previously reported [26]. Furthermore, the short variants were designed based on Pin2 [G] and Pin2 [GPG]. First, the Aspartic residue (D24) was replaced by a Lys in order to increase the net charge from +3 to +5. Second, the small hydrophobic (Ala) and the uncharged polar (Ser) residues were eliminated of the primary sequence to reduce the peptide length. To keep low hemolytic properties in the short CAMPs, the central Pro was replaced for Gly or for the GlyProGly triplet. Finally and prior to the chemical synthesis the amphipathic character of the resulted 14 and 17 amino acid sequences was optimized to values near to 50 and 30%, respectively. This was achieved by means of helical wheel projections (**Figure 1**), and by sequence search using 2,259 antimicrobial peptides with proved activity deposited in the APD2 database [39]. Also the amino acid sequence search had the goal to eliminate the casual probability to find characterized CAMPs similar to the designed short Pin2 variants. The physicochemical properties of Pin2 [G], Pin2 [GPG] and their short variants are shown in table 1. The amino acid sequences of the reported CAMPs with higher identity to Pin2 [14] and Pin2 [17] were aligned. Table 2 shows that the two short variants are only 40–54% similar to the parental peptide Pin2 and less than 50% similar to other natural and synthetic antimicrobial peptides.

**Table 2:** Alignment of the sequences of Pin2 [14] and Pin2 [17] with the sequence of other antimicrobial peptides.

Peptide	UniProtKB ID	Origin	Sequence‡	AA	Identity (%)	Q	Reference
Pin2 [14]	-	Synthetic	-FWG—LKGLKKFS---KKL--------	14	-	+5	This work
Pin2	P83240	*Pandinus imperator*	-FWGALAKGALKLI---PSLFSSFSKKD	24	50.0	+3	[15]
CPF-SE3	P84386	*Silurana epitropicalis*	GFLGSLLKTGLKVG---SNLL-------	18	50.0	+3	[46]
CE-MA§	-	Synthetic	-KWK—LFKKIKFLHSAKKF--------	17	47.4	+7	[47]
Macropin 1		*Macropis fulvipes*	---G—-FGMALKLL---KKVL-------	13	47.1	+3	[48]
Mastoparam	P69034	*Mischocyttarus phthisicus*	INW---LKLGKKMM---SAL--------	14	47.1	+3	[49]
Japonicin 1	B3VZU2	*Rana chensinensis*	-FFP—LALLCKVF---KKC--------	14	46.7	+3	[50]
Pin2 [17]		Synthetic	-FWG-LKGLKGP------GKFSKKL	17	-	+5	This work
Pin2	P83240	*Pandinus imperator*	-FWGALAKGALKLIPSLFSSFSKKD	24	54.2	+3	[15]
Ponericin W5	P82427	*Pachycondyla goeldii*	-FWGALIKGAAKLIPSVVGLFKKKQ	24	50.0	+5	[23]
CPF-SE3	P84386	*Silurana epitropicalis*	GFLGSLLKTGLK-----VGSNLL—	18	47.6	+3	[46]
NRC-15	-	*Glyptocephalus cynoglossus L*	GFWGKLFKLGLHG----IGLLHLHL	21	47.6	+2	[51]
Brevinin 1RTb	D1MIZ6	*Amolops ricketti*	-FLGSLLGLVGKVVPTLFCKISKKC	24	45.8	+4	[52]
Brevinin 1AUa	-	*Rana aurora aurora*	-FLPILAGLAAKLVPKVFCSITKKC	24	44.0	+4	[53]
Maxinin H39	Q58T55	*Bombina maxima*	-ILGPVLGLVGNAL----GGLIKKL	20	42.9	+2	[54]

‡ **, The sequence alignment was obtained using the on line program ClustawlW2 (** http://www.ebi.ac.uk/Tools/msa/clustalw2/ **).**

**AA, Number of amino acids.**

**Identity (%), sequence identity in percentage.**

**Q, Net Charge.**

**§, Cecropin A (residues 1–8)/Magainin 2b (residues 4–12) hybrid.**

### Peptide chemical synthesis and purification

**Figure 2:**
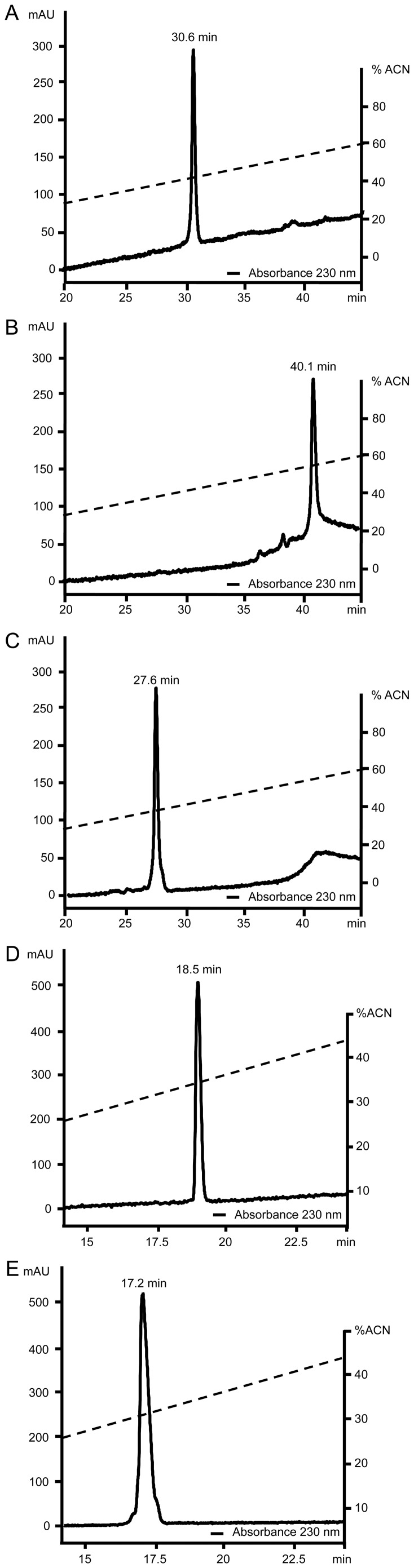
RP-HPLC purification of Pin2 and the variants characterized in this report. A. Pin2, B. Pin2 [G], C. Pin2 [GPG], D. Pin2 [14] and E. Pin2 [17].

The chemical synthesis of Pin2 and its variants was performed using a 0.1 mmol Fmoc chemistry. The crude synthetic peptide Pin2 and its variants were uniformly purified by RP-HPLC. As a result of the amino acid substitution and deletions in the primary structure of Pin2, the elution times of the Pin2 variants under RP-HPLC were shorter compared to the parental peptide except for Pin2 [G] (**Figure 2**). All purified Pin2 variants were proved to have the expected molecular masses. For instance, the experimental *versus* the theoretical masses expected were 2,612.0/2,612.1 for the native Pin2, 2,572.0/2,572.0 for Pin2 [G], 2,727.0/2,726.2 for Pin2 [GPG], 1,680.0/1,680.1 for Pin2 [14], and 1,891.3/1,891.3 for Pin2 [17]. As a consequence of the substitution and deletions in the primary structure of Pin2, the retention times (RT) under RP-HPLC changed, for instance the Pin2 [G] variant showed a RT of 40.1 min indicating a more hydrophobic molecule respect to Pin2 (RT of 30.6 min). Also, the less hydrophobic character of Pin2 [GPG] variant showed a RT of 27.6 min, while the RT of the short variants Pin2 [14] and Pin2 [17] were 18.5 and 17.2 min, respectively. The hydrophobic nature of the Pin2 variants observed by the experimental RT under RP-HPLC agreed to the theoretical hydrophobic character calculated using the GRAVY values. The amino acid sequences and some physicochemical properties of these peptides are listed in table 1. Once confirmed the identity of all synthesized peptides, their secondary structure was analyzed as well as their antimicrobial and hemolytic properties were observed.

### Circular dichroism secondary structure analysis

**Figure 3:**
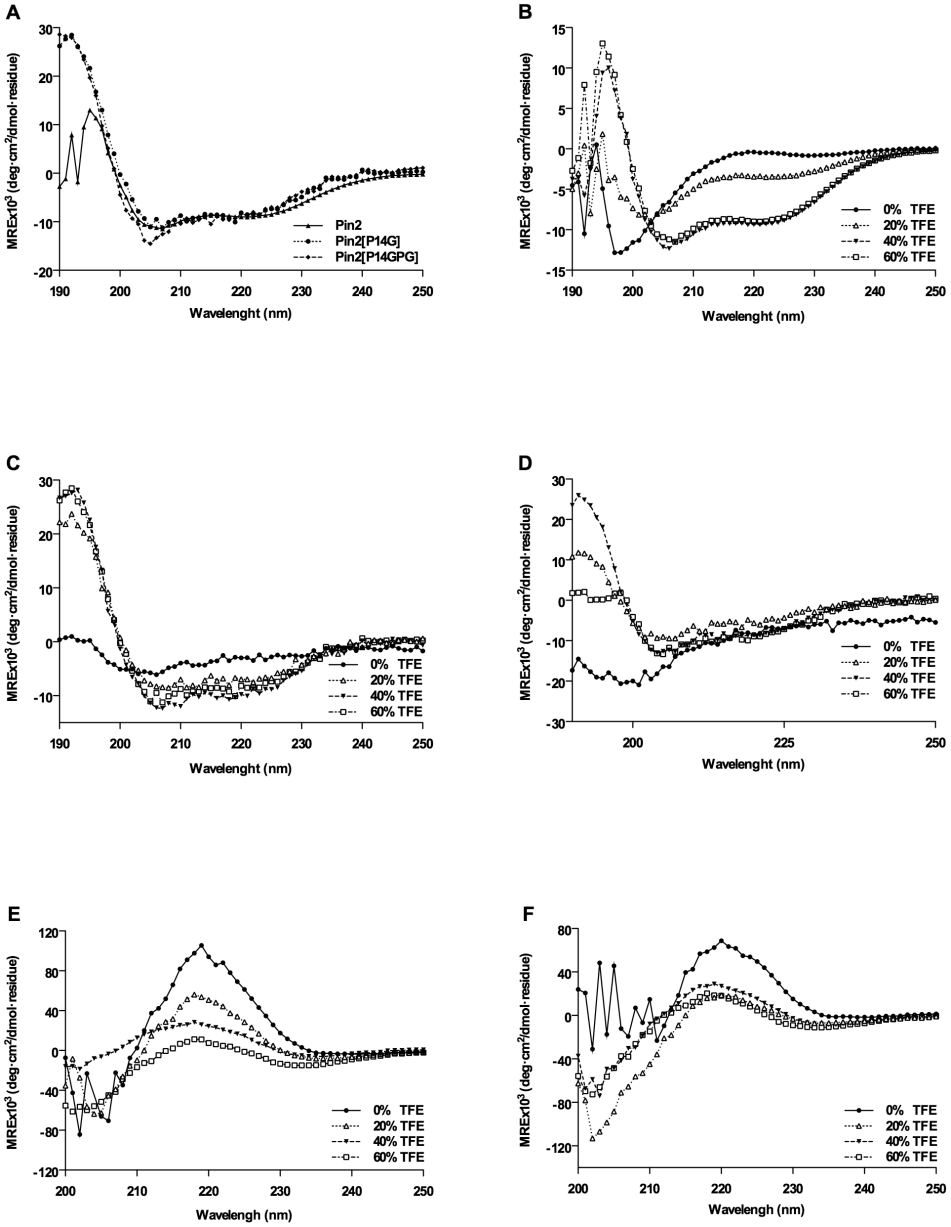
Circular dichroism of Pin2 and its long and short variants at different concentrations of TFE. A. CD spectra of Pin2, Pin2 [G] and Pin2 [GPG] at 60% TFE, B. Pin2, C. Pin2 [G], D. Pin2 [GPG], E. Pin2 [14], F. Pin2 [17].

The CD spectrum of Pin2 was compared to the CD spectra of the Pin2 [G] and Pin2 [GPG] variants. The secondary structure analysis was performed using CD spectral data from 190 to 260 nm, in the presence of 60% aqueous TFE, the CD spectra of Pin2 [G] and Pin2 [GPG] showed a clear ordered structure with two minimum ellipticity values at 208 and 222 nm (**Figure 3A**), indicating an alpha-helix conformation. In order to obtain more information concerning the propensity of these peptides to adopt alpha-helical structures, their CD spectra at different TFE proportions (0, 20, 40 and 60%) was acquired. The parental peptide Pin2 (**Figure 3B**) and the Pin2 [GPG] (**Figure 3D**) variant showed a clear “*random coil*” CD profile in the absence of TFE, but interestingly, the Pin2 [G] variant showed a 20% of alpha-helical structure in the absence of TFE (**Figure 3C**), suggesting a more structured Pin2 [G]. Pin2 [G] and Pin2 [GPG] adopted a maximum of 60% of alpha-helical structure at 40% TFE. Similarly, the CD spectra of the sort variants Pin2 [14] and Pin2 [17] were conducted at 0, 20, 40 and 60% TFE (**Figures 3E and 3F**). In the absence of TFE both Pin2 [14] and Pin2 [17] had clear positive ellipticities around 220 nm, and the increment in the TFE proportion induced a reduction in their ellipticity values. Furthermore, in the absence of TFE Pin2 [14] showed a strong negative ellipticity at 205 nm, and the increase of TFE enhanced its CD ellipticity value. Similar behavior was observed for Pin2 [17]. The CD deconvolution for Pin2 [14] and Pin2 [17] resulted in unstructured peptides even at the highest TFE concentration of 60%.

### Antimicrobial activities in Mueller-Hinton Agar and Mueller-Hinton Broth

The Gram-positive bacterium *S. aureus* is one of the main concerns in several topical infections while the Gram-negative bacterium *E. coli* is present in many gastrointestinal infections and other infection diseases. In agar culture media, the antimicrobial activity of Pin2 and Pin2 variants were tested against *S. aureus* and *E. coli*. Pin2 [G] variant displayed the highest antimicrobial activity with a MIC value of 12.5 µM, towards both *S. aureus* and *E. coli* (**Table 3**). Both parental Pin2 and Pin2 [GPG] showed MIC values of 37.5 and 25 µM for *S. aureus*, and 18.8 and 25 µM for *E. coli*, respectively. However the short variants Pin2 [14] and Pin2 [17] had MIC values >300 and 80 µM towards *S. aureus*, respectively, but they both Pin2 [14] and Pin2 [17] were more active towards *E. coli* with MIC values of 25 µM.

**Table 3:** Antimicrobial and hemolytic activities of Pin2 and the Pin2 variants.

		MIC (µM)		
Peptide	Assay	*E. coli*	*S. aureus*	Hemolysis (IC_50_)‡
Pin2	MHA	18.8	37.5	3.3 [1.9–5.7]
	MHB	12.5	12.5	
Pin2 [G]	MHA	12.5	12.5	1.4 [0.4–4.3]
	MHB	12.5	12.5	
Pin2 [GPG]	MHA	25	25	46.6 [34–64]*
	MHB	12.5	12.5	
Pin2 [14]	MHA	25	>300	418.4 [291–602]*
	MHB	25	>25†	
Pin2 [17]	MHA	25	80	NO
	MHB	>25†	>25	

MHA, Mueller-Hinton Agar.

MHB: Mueller Hinton Broth.

‡ Mean and 95% confidence intervals, values expressed in µM.

*The numeric IC_50_ value was obtained from the Boltzmann sigmoid equation fit.

† Bacteriostatic effect.

NO means no observed hemolytic activity at 100 µM.

**Figure 4:**
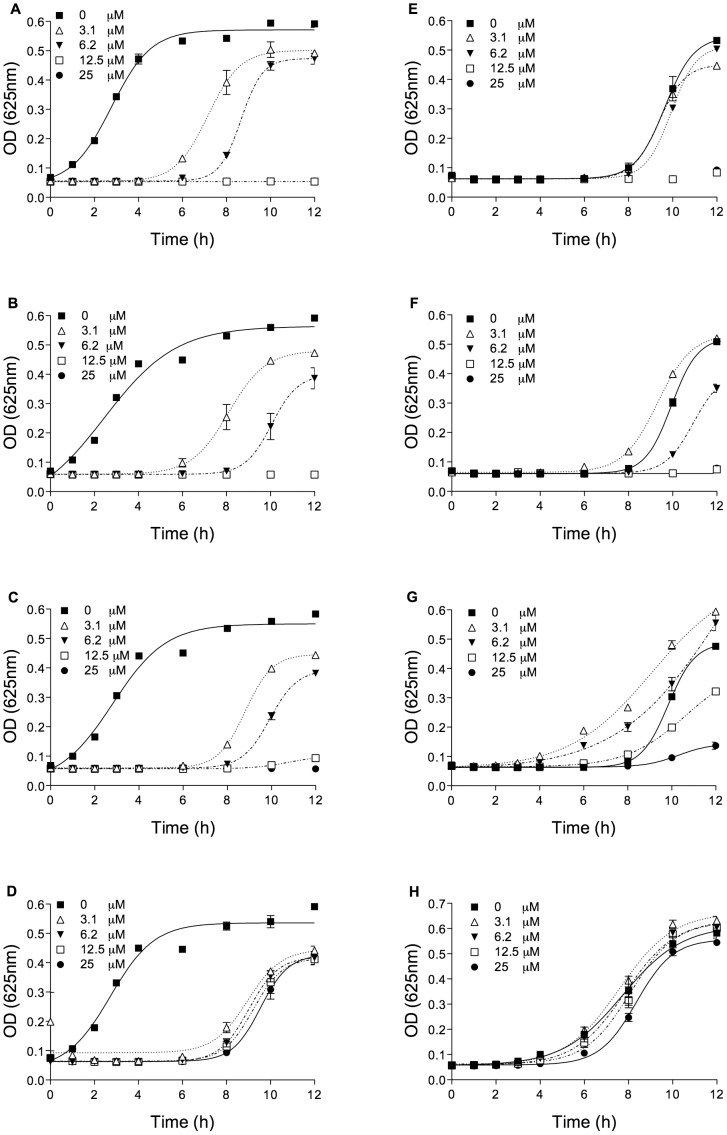
Antimicrobial activity of the Pin2 variants against *E. coli* ATCC 25922 and *S. aureus* ATCC 25923. *E. coli* antimicrobial activity, A. Pin2 [G], B. Pin2 [GPG], C. Pin2 [14], and D. Pin2 [17]. *S. aureus* antimicrobial activity, E. Pin2 [G], F. Pin2 [GPG], G. Pin2 [14], and H. Pin2 [17]. The concentration of peptides used was from 0.4 to 25 µM (n = 3).

In broth culture media, the bacterial growth of both *S. aureus* and *E. coli* was observed in the presence of serial concentrations of all Pin2 variants from 25 to 0.4 µM (**Figure 4**). Most of the synthetic peptides showed bactericidal or bacteriostatic antimicrobial activity against the two strains with MIC values of 12.5 and 25 µM. Pin2 [G] and Pin2 [GPG] showed MIC values of 12.5 µM for both *E. coli* and *S. aureus* (**Figures 4A, 4B, 4E and 4F**). The short variant Pin2 [14] had a MIC value of 25 µM against *E. coli* (**Figure 4C**) but a bacteriostatic effect at 25 µM against *S. aureus* (**Figure 4G**). Finally, the short variant Pin2 [17] only showed bacteriostatic effect on *E. coli* at 25 µM (**Figure 4D**), and no antimicrobial activity towards *S. aureus* was observed (**Figure 4H**). **Table 3** summarizes the MIC values observed for Pin2 and its synthetic variants.

### Activity of Pin2 variants against M. tuberculosis

The antibiotic capacities of Pin2 variants were tested in two strains of *M. tuberculosis* using the REMA methodology. *M. tuberculosis* H37Rv is an ATCC strain which has been widely studied and its genome have been completely sequenced, on the other hand, *M. tuberculosis* MDR is a clinical isolated strain which possesses antimicrobial resistance against rifampicin and isoniazid, which are antibiotics used commercially for the treatment of tuberculosis. All synthetic peptide variants showed antimicrobial activities over both strains of *M. tuberculosis* (**Table 4**). The MIC values observed were from 11 to 30 µM for the *M. tuberculosis* H37Rv and from 6 to 33 µM for *M. tuberculosis* MDR. It was interesting to observe that the two short variants Pin2 [14] and Pin2 [17] had MIC values of 11.9 and 11.6 µM towards *M. tuberculosis* H37Rv and MIC values of 6 and 14.8 µM against *M. tuberculosis* MDR, respectively. These results show the feasibility for using short sequence CAMPs against pathogenic *M. tuberculosis* strains that have acquired resistance to commercial antibiotics.

**Table 4:** Antimicrobial activity of Pin2 and the Pin2 variants on two strains of *M. tuberculosis*.

		*Mycobacterium tuberculosis* H37Rv		*Mycobacterium tuberculosis* MDR*	
Peptide	MW (Da)	MIC (µg/mL)	MIC (µM)	MIC (µg/mL)	MIC (µM)
Pin2	2,612.1	57.7±22.3	22.1±8.6	86.5	33.1
Pin2 [G]	2,572.0	48.1	18.7	48.1	18.7
Pin2 [GPG]	2,762.2	80.1±24.8	29±9	48.1	17.4
Pin2 [14]	1,680.1	20±6.2	11.92±3.7	10±3.1	6±1.8
Pin2 [17]	1,891.3	22±4.9	11.65±2.59	28.0±9.8	14.8±5.2
Ethambutol§	204.3	0.5	2.5	20	97
Isoniazid	137.1	24±8.8	175.1±63.9	6±2.2	43.8±16
Rifampicin§	823.0	0.4	0.5	32	38.9

**The MIC values were calculated using the Resazurin dye reduction method, 500,000 bacteria per well were evaluated.**

**§, MIC values reported by Rastogi, et al., 1996** [55] **.**

*, Clinically isolated strain characterized with resistance to rifampicin and isoniazid in UIMZ-IMSS, Zacatecas, Mexico.

### Hemolytic activity of Pin2 variants

**Figure 5:**
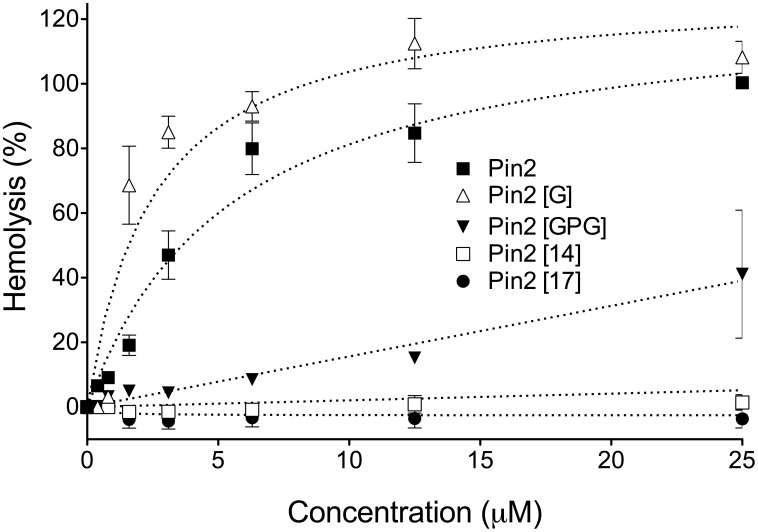
Hemolytic activity in human red blood cells. Data are the average of at least four independent experiments. Error bars represent the standard deviations.

The hemolytic assays of Pin2 and the Pin2 variants on human erythrocytes (**Figure 5**) showed that the parental peptides Pin2 as the Pin2 [G] variant at 25 µM had the highest hemolytic activities of all five peptides evaluated, with a 91% and a 100% of hemolysis, respectively. However at the same concentration the Pin2 [GPG] variant showed only a 30% of hemolysis, indicating that the insertion of the GPG motif is relevant for hemolytic activity reduction. The two short variants had not hemolytic effects at 25 µM. Furthermore, they were assayed up to 100 µM observing that the peptide Pin2 [14] showed 25% of hemolysis, while Pin2 [17] did not show any hemolytic effect at such concentration. The statistical analysis is shown in table S1 in file S1.

## Discussion

Proline residues are commonly founded in the central regions of alpha-helical segments of transmembrane proteins [34], [35]. Similarly to these proteins, proline plays an important role in the biological activity of CAMPs, where it imparts significant structural and functional properties [36]. For example, melittin, gaegurins, pardaxin, pipinins, brevinins and ponericins contain a proline residue in the central region of their sequences (**Table S2 in file S1**). Several reports had mentioned that this residue gives a higher capacity to develop pores in bacterial cell membranes, such characteristic is important for their antimicrobial activities, but also CAMPs containing Pro show high hemolytic activities at low concentrations, indicating a low selectivity between bacterial and mammalian cell membranes [17], [19]–[21]. As reported here, the proline residue provides hemolytic properties to Pin2; therefore, to reduce its hemolytic properties, it was substituted by glycine, but unexpectedly Pin2 [G] kept its hemolytic properties; so, also it was flanked by two glycine residues in order to reduce their lytic activities on mammalian cells but to maintain its antimicrobial properties. Additionally, two short peptides were designed to reduce the length of Pin2. As a consequence of the substitution and deletions in the primary structure of Pin2, the physicochemical properties of the Pin2 variants changed (**Table 1**).

The CD spectrum of Pin2, Pin2 [G] and Pin2 [GPG] (**Figure 3B–D**) in absent of TFE was typical of unstructured proteins or random coil proteins. However, all three peptides Pin2, Pin2 [G] and Pin2 [GPG] showed classic α-helical structures at 60% TFE (**Figure 3B–D**) showing two negative bands of similar magnitude at 222 and 208 nm, and a positive band in the range from 190 to 200 nm typical of structured α-helical proteins [37], [38]. These results suggest a high propensity to form α-helical structures in hydrogen promoting solvents, which mimics cell membrane environments. On the other hand, the CD profiles of the short peptides were unexpected even at 60% of TFE; for example, Pin2 [14] in the absence of TFE had a strong positive band displayed around 220 nm and a negative band around 205 nm (**Figure 3E**). The positive band could be related to the presence of the aromatic side chains of phenylalanine and tryptophan residues (exciton coupling) in the amine termini region of this peptide, and the negative band near to 205 nm indicates a random coil conformation [39], [40]. The increment of the TFE proportion resulted in reduction of the positive band but the negative band, thus indicating the secondary structure induction, similar as was observed by Gopal *et al*. (2012) with the (KW)_4_ peptide [41]. Nevertheless, since the proposed antimicrobial peptide mechanism of action occurs because structural peptide arrangements with the bacterial membrane, the CD structural modifications of Pin2 [14] may explain its antimicrobial activity. The presence of positive bands observed around at 220 nm for Pin2 [17] (**Figure 3F**) could be explained also for the same aromatic chromophore effects in Pin2 [14]. However the increment in the negative band around 203 nm, resulted for the TFE increments, could be related to a beta-hairpin structural conformation, similar to the indolicidin, a poliproline antimicrobial peptide having tryptophan residues in its primary structure [42] and also because of the presence of structure disruptor residues, such as, the presence of the GlyProGly tripled in the middle of its structure and the three additional glycine residues, Gly6, Gly9 and Gly11. Similar CD profiles compared with those of the Pin2 [17] were observed by Riemen and Waters (2010) with WSWS peptide series. These 12 mer peptides have an exciton coupling of the aromatic residues Trp2 and Trp9. For example, the WSWS peptide RWVSVNGKWISQ has a CD spectrum containing a minimum at 215 nm and a maximum at 229 nm [43]. In this report, Pin2 [17] contains the aromatic residues phenylalanine and tryptophan in close distance that could result in a minimum at 204 nm and a maximum at 220 nm; so the observed CD spectrum could be also the consequence of a random coil conformation with an aromatic exciton coupling similar to that observed for the WSWS peptides.

The antimicrobial activities of Pin2 [G] and Pin2 [GPG] were from 12.5 to 25 µM, in both MHA and MHB bacterial cultures, towards *E. coli* and *S. aureus*. Although the antimicrobial activities of Pin2 [G] and Pin2 [GPG] were quite similar to the parental peptide, the antimicrobial activity of Pin2 [G] was better than that of Pin2 [GPG] in both MHA and MHB bacterial cultures. Concerning the hemolytic activities, Pin2 [G] was more hemolytic that the parental peptide. This increase in function in Pin2 [G] might be related to its more hydrophobic character and to its more ordered alpha-helical structure. With respect to the antimicrobial activity of the short variants, Pin2 [14] and Pin2 [17], its antimicrobial activities were acceptable towards *E. coli* in both MHA and MHB (25 µM), but very poor towards *S. aureus*. However, they were less lytic to erythrocytes.

In order to correlate the differences observed in the hemolytic activity of the different variants with their amphipathicity, we look at the helical wheel projections of Pin2 and Pin2 variants (**Figure 1**). First, both helical wheels of Pin2 and Pin2 [G] look similar with the exception that substitution of the proline residue by glycine. This substitution did not introduce a modification in the amphipathycity of the parental peptide Pin2 (**Figure 1A–B**); however, for Pin2 [GPG] it is observed that the hydrophilic part of this peptide is crowded with hydrophobic residues (**Figure 1C**, left side). Here, it is also observed that the hydrophobic face (**Figure 1C**, right side) has been disrupted because of the inclusion of the hydrophilic residues Gly15 and Ser17. Nevertheless, the residues Ser20 and Ser23 in the hydrophobic face may balance the amphipathic properties of Pin2 [GPG]. Such hydrophobic balance between the left and right side observed in the helical wheel seems to cause a reduction in the hemolytic activity of this peptide. Concerning the helical wheel projection of the short variants, these representations show the amphipathic distribution as they were previously designed, so the diminish in the hemolytic profile of the short variants might be related to the reduction of hydrophobic residues. These results could be related to the hemolytic activity assays, for instance, the peptide with more alpha-helix propensity and more hydrophobic, Pin2 [G], showed the highest antimicrobial and hemolytic activity, while, the peptide with the less pronounced alpha-helix propensity and less hydrophobic Pin2 [GPG], at the same time showed a reduction in its antimicrobial and hemolytic activity. Respect to the short Pin2 variants, the CD data analysis indicates that Pin2 [14] and Pin2 [17] are beta-structured, contrary to the predicted structure following the Schiffer-Edmundson wheel projections. The non-helical secondary structure observed in both Pin2 [14] and Pin2 [17] was not an impediment to exert antimicrobial activities. Although they were lower to their helical derived peptides for the Gram-positive and Gram-negative bacteria, Pin2 [14] and Pin2 [17] were more efficient to inhibit the growth of *Mycobacterium tuberculosis*.

Concerning the activity against *M. tuberculosis*, it is notable that the larger peptides Pin2, Pin2 [G] and Pin2 [GPG] had lower inhibitory capacity than the shorter peptides Pin2 [14] and Pin2 [17]. Also, it is noteworthy that Pin2 and its variants are comparable to the antibiotics used for the treatment of tuberculosis (i.e. ethambutol, rifampicin, isoniazid) at the molar doses. This comparison is more evident in the inhibition of *M. tuberculosis* MDR, and the interest to design short antimicrobial peptides.

Here we based the design of low hemolytic and short antimicrobial peptides on observed patterns in nature and on theoretical calculations. We found a strong correlation in hydrophobicity and alpha-structured molecules with high hemolytic activity; however, the antimicrobial capacity could be sustained with low eukaryotic lytic activities in short hydrophilic antimicrobial peptides such that Pin2 [14] and Pin2 [17] with a plus of maintaining a wider antimicrobial spectrum; that is affecting Gram-positive and Gram-negative bacteria as well as multi-drug resistant *Mycobacterium tuberculosis*.

## Supporting Information

File S1 **Supporting tables.** **Table S1**, Statistical analysis of variance with ANOVA of the hemolysis data, followed by post hoc testing using the Tukey's method. **Table S2**, Proline in the middle regions of hemolytic antimicrobial peptides.
(DOCX)
