# Supplementary material for: Characterization of Antibacterial and Hemolytic Activity of Synthetic Pandinin 2 Variants and Their Inhibition against Mycobacterium tuberculosis
Source: PLoS One. 2014 Jul 14;9(7):e101742. doi: 10.1371/journal.pone.0101742 (PMC4096598; doi:10.1371/journal.pone.0101742)
Supplement: File S1 — Supporting tables. Table S1, Statistical analysis of variance with ANOVA of the hemolysis data, followed by post hoc testing using the Tukey's method. Table S2, Proline in the middle regions of hemolytic antimicrobial peptides. (DOCX) [file pone.0101742.s001.docx]

**Table S1. Statistical analysis of variance with ANOVA of the hemolysis data, followed by post hoc testing using the Tukey’s method.**

| Means comparison ANOVA | |  | Significance P <0.05* |
| --- | --- | --- | --- |
| Pin2 | Pin2 [G] |  | P>0.05 |
| Pin2 | Pin2 [GPG] |  | P<0.05 |
| Pin2 | Pin2 [14] |  | P<0.05 |
| Pin2 | Pin2 [17] |  | P<0.01 |
| Pin2 [G] | Pin2 [GPG] |  | P<0.01 |
| Pin2 [G] | Pin2 [14] |  | P<0.001 |
| Pin2 [G] | Pin2 [17] |  | P<0.001 |
| Pin2 [GPG] | Pin2 [14] |  | P>0.05 |
| Pin2 [GPG] | Pin2 [17] |  | P>0.05 |
| Pin2 [14] | Pin2 [17] |  | P>0.05 |
| *Tukey’s Multiple Comparison Test (GraphPad, Inc., USA)  Pin2^a^; Pin2 [G]^a^; Pin2 [GPG]^b^ ; Pin2 [14]^b^; Pin2 [17]^b^ | | | |

**Table S2. Proline in the middle regions of hemolytic antimicrobial peptides**

| Peptide | Sequence | Identity (%) | Reference |
| --- | --- | --- | --- |
| Pandinin 2 | FWGALAKGALKLI**P**SLFSSFSKKD-- | 100.0 | [[1](#_ENREF_1)] |
| Brevinin 1 | FLPVLAGIAAKVV**P**ALFCKITKKC-- | 75.2 | [[2](#_ENREF_2)] |
| Gaegurin 5 | FLGALFKVASKVL**P**SV KCAITKKC- | 73.2 | [[3](#_ENREF_3)] |
| Pipinin 1 | FLPIIAGVAAKVF**P**KIFCAISKKC-- | 72.2 | [[4](#_ENREF_4)] |
| Pipinin 2 | FLPIIAGIAAKVF**P**KIFCAISKKC-- | 71.4 | [[4](#_ENREF_4)] |
| Ponericin W5 | FWGALIKGAAKLI**P**SVVGLFKKKQ-- | 70.0 | [[5](#_ENREF_5)] |
| Gaegurin 6 | FLPLLAGLAANFL**P**TIICKISYKC-- | 67.0 | [[3](#_ENREF_3)] |
| Melittin | GIGAVLKVLTTGL**P**ALISWIKRKRQQ | 63.4 | [[6](#_ENREF_6)] |
| Ponericin W3 | IWGTLAKIGIKAV**P**RVISMLKKK--- | 56.0 | [[5](#_ENREF_5)] |

**References of supporting information**

1. Corzo G, Escoubas P, Villegas E, Barnham KJ, He W, et al. (2001) Characterization of unique amphipathic antimicrobial peptides from venom of the scorpion Pandinus imperator. Biochem J 359: 35-45.

2. Morikawa N, Hagiwara Ki, Nakajima T (1992) Brevinin-1 and -2, unique antimicrobial peptides from the skin of the frog, *Rana brevipoda porsa*. Biochem Biophys Res Commun 189: 184-190.

3. Park JM, Jung JE, Lee BJ (1994) Antimicrobial Peptides from the Skin of a Korean Frog, Rana rugosa. Biochem Biophys Res Commun 205: 948-954.

4. Goraya J, Wang Y, Li Z, O'Flaherty M, Knoop FC, et al. (2000) Peptides with antimicrobial activity from four different families isolated from the skins of the North American frogs *Rana luteiventris*, *Rana berlandieri* and *Rana pipiens*. Eur J Biochem 267: 894-900.

5. Orivel J, Redeker V, Le Caer J-P, Krier F, Revol-Junelles A-M, et al. (2001) Ponericins, new antibacterial and insecticidal peptides from the venom of the ant *Pachycondyla goeldii*. J Biol Chem 276: 17823-17829.

6. Kreil G (1973) Structure of melittin isolated from two species of honey bees. FEBS Lett 33: 241-244.
